# Geospatial Hot Spots and Cold Spots in US Cancer Disparities and Associated Risk Factors, 2004–2008 to 2014–2018

**DOI:** 10.5888/pcd21.240046

**Published:** 2024-10-31

**Authors:** L. Raymond Guo, M. Courtney Hughes, Margaret E. Wright, Alyssa H. Harris, Meredith C. Osias

**Affiliations:** 1 College of Health and Human Sciences, Northern Illinois University, DeKalb; 2 University of Illinois Cancer Center, Chicago

## Abstract

**Introduction:**

Despite declining cancer death rates in the US, cancer remains the second deadliest disease and disparities persist. Although research has focused on identifying risk factors for cancer deaths and associated disparities, few studies have examined how these relationships vary over time and space. The primary objective of this study was to identify cancer mortality hot spots and cold spots — areas where cancer death rates decreased less than or more than neighboring areas over time. A secondary objective was to identify risk factors of cancer mortality hot spots and cold spots.

**Methods:**

We analyzed county-level cancer death rates from 2004 through 2008 and 2014 through 2018, exploring disparities in changes over time for socioeconomic and demographic variables. We used hot spot analysis to identify areas with larger decreases (cold spots) and smaller decreases (hot spots) in cancer death rates and random forest machine learning analysis to assess the relative importance of risk factors associated with hot spots and cold spots. We mapped spatial clustering areas.

**Results:**

Geospatial analysis showed hot spots predominantly in the Plains states and Midwest and cold spots in the Southeast, Northeast, 2 Mountain West states (Utah and Idaho), and a portion of Texas. Factors with the strongest influence on hot spots and cold spots were unemployment, preventable hospital stays, mammography screening, and high school education.

**Conclusion:**

Geospatial disparities in changes in cancer death rates point out the critical role of access to care, socioeconomic position, and health behaviors in persistent cancer mortality disparities. Study results provide insights for interventions and policies that focus on addressing health care access and social determinants of health.

**Summary**

**What is already known on this topic?**

Disparities in cancer death rates exist across the social gradient, with lower socioeconomic groups and racial and ethnic minority populations experiencing higher death rates.

**What is added by this report?**

We used geospatial analysis to identify hot spots and cold spots of disparities in cancer death rates across US counties. We identified factors associated with these disparities, including access to care, health behaviors, and social determinants of health.

**What are the implications for public health practice?**

Policy and interventions should address geospatial disparities, focusing on social determinants of health, health care access, and healthy behaviors to achieve equitable cancer outcomes.

## Introduction

Despite declines in cancer death rates during the past 3 decades, cancer remains the second leading cause of death in the US (1,2). Disparities in cancer death rates also persist across many groups in the US (1). For example, the decline in cancer death rates has been slower for groups with lower socioeconomic position and for racial and ethnic minority populations, particularly Black Americans. Cancer death rates also vary geographically: Southern states and rural areas of the country have the highest cancer death rates and slower rates of decline (3).

Social determinants of health (SDOH), defined as the conditions in which people live, work, and age (4,5), contribute to cancer death and persistent cancer-related disparities. SDOH domains include economic stability (eg, poverty, food insecurity), education access and quality, health care access and quality, neighborhood and built environment (eg, violence, air pollution), and social and community context (eg, social support) (4). SDOH can be beneficial or adverse, and they affect health outcomes through influence on health behaviors, environmental exposures, stress levels, and access to care.

Several studies have linked unfavorable SDOH, particularly lower education and income, to higher cancer death rates (3,6,7) and positive SDOH, such as access to cancer care (8), private health insurance (9), and access to healthy diets (10), to better cancer outcomes. Like cancer death rates, SDOH and downstream risk factors (eg, smoking, physical activity, and diet) vary by geography.

Few studies have examined how relationships among SDOH and cancer death rates vary over time and by geography (11). Understanding these dynamic relationships is crucial for adequately and accurately addressing persistent cancer disparities and identifying targets for intervention and resource allocation. The primary objective of this study was to identify cancer mortality hot spots and cold spots — areas where cancer death rates decreased less than or more than neighboring areas over time. A secondary objective was to identify risk factors of cancer mortality hot spots and cold spots.

## Methods

We obtained age-adjusted total cancer death rates at the county level, available in 5-year aggregates from 2004 through 2018, from CDC WONDER (Centers for Disease Control and Prevention Wide-ranging ONline Data for Epidemiologic Research) (12). National mortality data available on CDC WONDER are publicly available; data are collected by state registries and provided to the National Vital Statistics System. Data are based on death certificates for US residents; each death certificate specifies a single underlying cause of death and includes demographic data. The number of deaths and death rates can be obtained at multiple geographic levels (national, state, and county, when available), and by age group, race, Hispanic ethnicity, sex, and cause of death (4-digit ICD-10 [*International Classification of Diseases, Tenth Revision*] codes [13]). We collected the data from the US 1999–2018: Underlying Cause of Death data file in CDC WONDER and used ICD-10 codes C00–C97 to identify death rates for malignant neoplasms.

Next, we identified risk factors for total cancer death rates by conducting a comprehensive review of the published literature in PubMed. We used the search terms “cancer mortality” AND (“risk factor” OR “determinants” OR “predictors”) for the period 2000 to 2022. Two reviewers (L.R.G., M.C.O.) conducted the PubMed search, ensuring that the search and selection of studies were comprehensive and unbiased. Risk factors were identified through a qualitative meta-review or second-order review of the evidence associated with SDOH and cancer burden, focusing on systematic reviews and meta-analyses to synthesize and evaluate the literature comprehensively. This approach ensured that the collected data could be used for complex analysis methods, such as random forest or other machine learning models. We then obtained county-level data for identified risk factors from CDC (14), the US Census Bureau (15), and County Health Rankings & Roadmaps (16). These data allowed us to explore how risk factors might influence cancer deaths at the same geographic level as the CDC WONDER mortality data. Final data included age-adjusted total cancer death rates and aggregated individual risk factors, such as demographic factors, health behaviors, and SDOH and were used for both geospatial analysis and machine learning analysis (Table).

**Table:** Variables and definitions for Study of Cancer Disparities and Associated Risk Factors, United States, 2004–2018

Variable^a^	Definition^b^	Sources
**Total cancer mortality rate**	The number of deaths, with all types of cancer as the underlying cause of death, occurring in a specified population during a time frame.	CDC Wide-ranging ONline Data for Epidemiologic Research (WONDER) (12)
**Demographic characteristics**		
Age	Age of respondent, grouped as ≥65 or <65 years.	American Community Survey (15)
Race and ethnicity		
Non-Hispanic American Indian or Alaska Native	A person having origins in any of the original peoples of North and South America (including Central America) and who maintains tribal affiliation or community attachment.	American Community Survey (15)
Non-Hispanic Asian	A person having origins in any of the original peoples of the Far East, Southeast Asia, or the Indian subcontinent including, for example, Cambodia, China, India, Japan, Korea, Malaysia, Pakistan, the Philippine Islands, Thailand, and Vietnam.	American Community Survey (15)
Non-Hispanic Black or African American	A person having origins in any of the Black racial groups of Africa.	American Community Survey (15)
Hispanic	A person of Cuban, Mexican, Puerto Rican, South or Central American, or other Spanish culture or origin regardless of race.	American Community Survey (15)
Non-Hispanic Native Hawaiian and Other Pacific Islander	A person having origins in any of the original peoples of Hawaii, Guam, Samoa, or other Pacific Islands. This includes people who reported detailed Pacific Islander responses such as Native Hawaiian or Other Pacific Islander; Fijian; Chamorro; Marshallese; Native Hawaiian; Other Micronesian; Other Pacific Islander; not Specified; Other Polynesian; Samoan; and Tonga.	American Community Survey (15)
Non-Hispanic White	A person having origins in any of the original peoples of Europe, the Middle East, or North Africa.	American Community Survey (15)
Sex	Respondents mark either male or female to indicate their biological sex.	American Community Survey (15)
**Health behaviors**		
Smoking	Percentage of adults that reported currently smoking.	Behavioral Risk Factor Surveillance System (14)
Obesity	Percentage of adults that report having a body mass index ≥30.	Behavioral Risk Factor Surveillance System (14)
Physical inactivity	Percentage of adults that report no leisure-time physical activity.	County Health Rankings & Roadmaps (16)
Drinking	Percentage of adults that report excessive drinking.	Behavioral Risk Factor Surveillance System (14)
**Access to care, health literacy, health conditions**		
Lacks health insurance	Respondents who do not have health insurance coverage as from private health insurance or public coverage	County Health Rankings & Roadmaps (16)
High school education	Respondents who received at least a regular high school diploma and did not attend college were instructed to report “regular high school diploma.”	County Health Rankings & Roadmaps (16)
Unemployment	Respondents aged ≥16 years who were neither “at work” nor “with a job but not at work” during the reference week and were actively looking for work during the last 4 weeks, and were available to start a job.	County Health Rankings & Roadmaps (16)
Mammography screening	Percentage of female Medicare enrollees having ≥1 mammogram in 2 years.	County Health Rankings & Roadmaps (16)
Preventable hospital stay	Discharges for ambulatory care sensitive conditions per 1,000 Medicare enrollees.	County Health Rankings & Roadmaps (16)
Primary care physicians	Primary care physicians per 100,000 population.	County Health Rankings & Roadmaps (16)
Poor health	Percentage of adults that report fair or poor health.	County Health Rankings & Roadmaps (16)
Poor health days	Average number of reported physically unhealthy days per month.	County Health Rankings & Roadmaps (16)
Poor mental health days	Average number of reported mentally unhealthy days per month.	County Health Rankings & Roadmaps (16)
Mental health providers	Mental health providers per 100,000 population.	County Health Rankings & Roadmaps (16)
**Economic stability**		
Housing problem	Percentage of households with at least 1 of 4 housing problems: overcrowding, high housing costs, lack of kitchen, lack of plumbing facilities.	County Health Rankings & Roadmaps (16)
Household without vehicle	Percentage of households that do not own ≥1 vehicle.	County Health Rankings & Roadmaps (16)
Median household income	A measure that divides the selected monthly owner costs as a percentage of household income distribution into 2 equal parts: one-half of the cases falling below the median selected monthly owner costs as a percentage of household income and one-half above the median	County Health Rankings & Roadmaps (16)
Transportation	Percentage of housing units with no vehicle available.	County Health Rankings & Roadmaps (16)

a Total cancer death rate is the dependent variable; all others are independent variables.

b Definitions are from original data sources.

Ethics approval and consent to participate were not applicable in this study as decided by Northern Illinois University Institutional Review Board. This secondary analysis used publicly available datasets.

### Study variables

Our study was designed to identify hot spots and cold spots of county-level cancer mortality changes between 2004–2008 and 2014–2018 based on available data. We used these periods to focus on longer trends and examined negative SDOH and demographic risk factors linked to persistent disparities over time (Table). Total cancer mortality was the dependent variable, while demographic characteristics, health behaviors, access to care, health literacy, health conditions, and economic stability were categorized as independent variables.

### Geospatial hot spot analysis

We calculated changes in total cancer death rates as the difference in the rate between 2004–2008 and 2014–2018 by subtracting the 2014–2018 rate from the 2004–2008 rate. We applied imputation to the counties that had missing data by taking the average of the surrounding counties. We then applied Getis-Ord Gi* analysis with the Euclidean distance as fixed distance in ArcGIS Pro 2.7 (Esri) (17). Euclidean distance measures the direct distance between 2 nearest counties to ensure each feature has at least 1 neighbor. We identified hot spots and cold spots on the changes in total cancer death rates. We linked CDC WONDER data with the data file “USA Counties, August 4, 2022 updated” in ArcGIS Pro 2.7 by the Federal Information Processing Standard (FIPS) code at the county-level GIS layer. The hot spot analysis examines each geographical feature in the context of neighboring features. It calculates *z* scores and *P* values to identify where features with either high or low values cluster spatially compared with neighboring areas. We set the significance level at .10 to capture more potential hot spots with a high prevalence of cancer. This significance level can help to find weak clustering patterns that may have some significance. For this study, we defined hot spots as counties with a significantly smaller decrease (*P* <.10) in cancer mortality rates between 2004–2008 and 2014–2018 compared with the averages of the cluster of surrounding counties. In contrast, cold spots refer to counties with a significantly larger decrease in total cancer mortality rates during the same period compared with the averages of the cluster of surrounding counties. Positive *z* scores indicate the clustering of high values, signifying a hot spot, while negative *z* scores indicate the clustering of low values, signifying a cold spot. A *z* score near zero suggests no apparent spatial clustering and can be considered an average area. We then mapped spatial clustering areas and identified hot spots and cold spots (18).

### Analysis of associated SDOH and downstream risk factors

First, we applied random forest analysis to address our second study objective to identify SDOH and downstream risk factors associated with hot spots and cold spots. Data on SDOH, demographic characteristics, and health behavior were available for 1,614 of all 3,143 US counties from 2004 to 2018. We used the variables as presented in the datasets. Previous studies with a comparable scope also faced challenges related to missing data and analyzed only a subset of the 3,143 counties (19–21).

Next, as part of random forest analysis, we selected variables by using a bagging technique that generated multiple bootstrap samples from the original dataset. Bagging is an ensemble learning technique that combines multiple models trained on bootstrapped subsets of the original dataset to improve predictive performance and reduce variance (22). We then used these bootstrap samples to train a multitude of decision trees, where each tree randomly selects features at each split point, creating a “forest” that votes on the final prediction. Each decision tree acts like a flowchart, splitting the data based on features (risk factors). We then calculated the importance of each predictor in making those predictions. This approach helped identify which predictor had the strongest influence on the outcome. By averaging importance scores from multiple decision trees, random forest analysis captures individual variable influence and interactions while reducing overfitting, enabling a more robust understanding of variables that have the most effect on changes in total cancer death rates at the county level (22). This ensemble approach strengthens the analysis by addressing variance in single-tree predictions, leading to a more robust understanding of which variables are truly important.

We applied the random forest algorithm to all 1,614 counties and the hot spot and cold spot clusters between 2004–2008 and 2014–2018 using 22 SDOH and other factors (Table) to determine their effect on changes in cancer death rates. The random forest model ranked the most important variable at 100% and scaled all other variables in relation to it. In this analysis, the more important the factor, the greater the effect on the model’s ability to predict cancer death rates. We used the “randomForest” package in R 4.2 (R Core Team, 2023) for these analyses.

## Results

**Figure 1:**
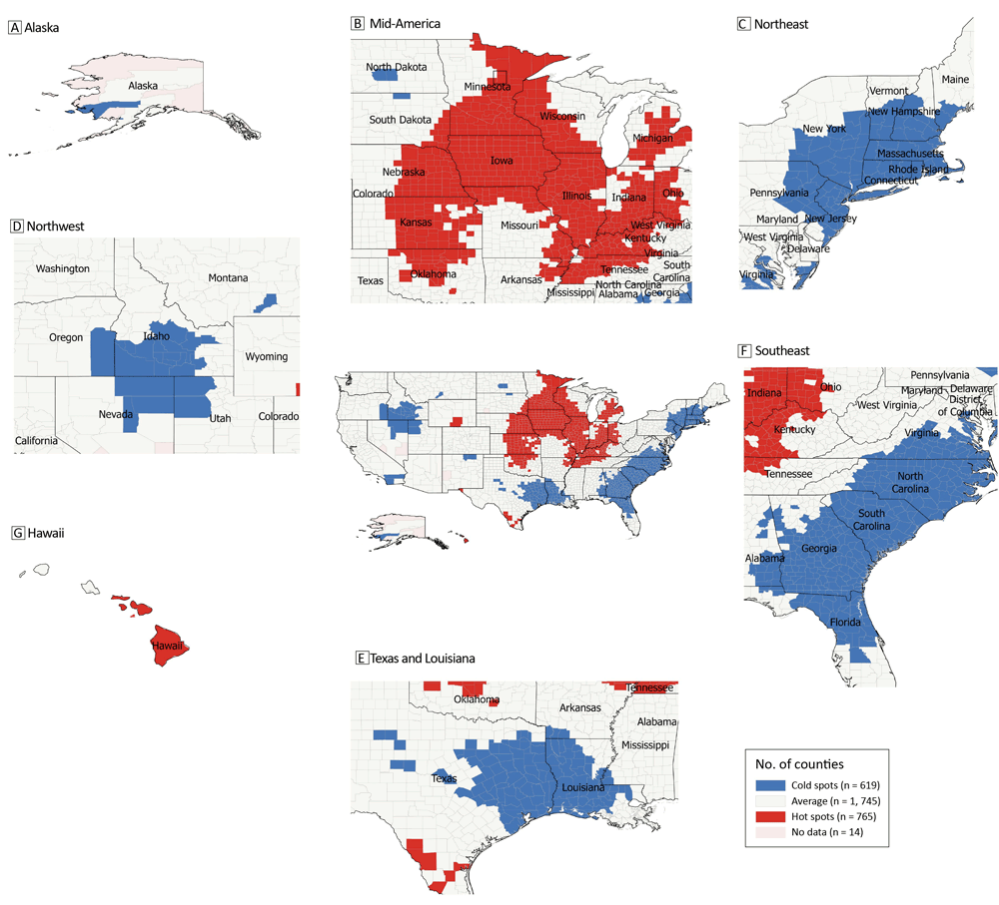
Changes in total cancer death rates at the county level between 2004–2008 and 2014–2018 in the US.

The hot spot analysis (Figure 1) showed a large cluster on the US mainland (primarily in the Plains states and the Midwest) and a few counties in Hawaii. Cold spots were located in the Southeast, a portion of the Northeast, 2 states in the Mountain West (Utah and Idaho), and portions of Texas, Louisiana, and Alaska. The mean (SD) change in total cancer death rates in the US was −21.23 (18.75) deaths per 100,000 people. The mean (SD) change in death rate per 100,000 people was −16.6 (18.8) deaths for hot spots and −25.5 (18.4) deaths for cold spots. The mean percentage change was −7.7% for hot spots and −12.8% for cold spots. Additionally, the CDC WONDER dataset has an average 18.7% missing death rate data for all counties and states each year. A few states have an average missing data rate higher than 35%: Texas (86.2%), Nebraska (56.2%), Kansas (51.1%), and South Dakota (39.7%). Other states that have missing data are Alaska (22.3%), Idaho (18.7%), Minnesota (11.8%), Missouri (13.9%), Oklahoma (12.9%), Utah (12.9%), and Virginia (13.3%).

### Risk factors for cancer mortality hot spots and cold spots

**Figure 2:**
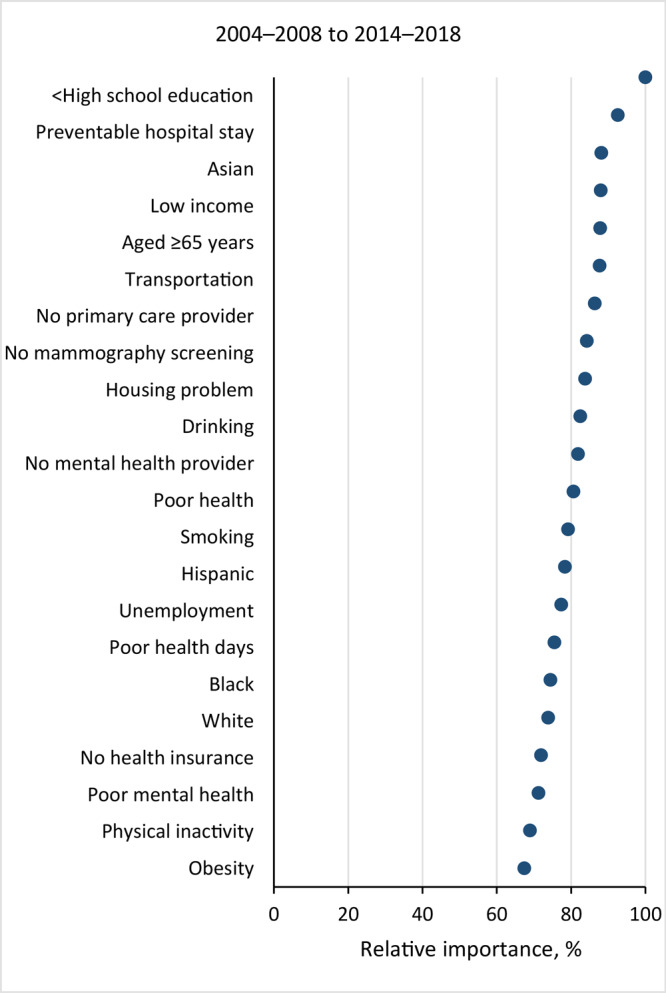
Relative importance of predictors of changes in total cancer mortality rates between 2004 and 2018 in US counties.

**Figure 3:**
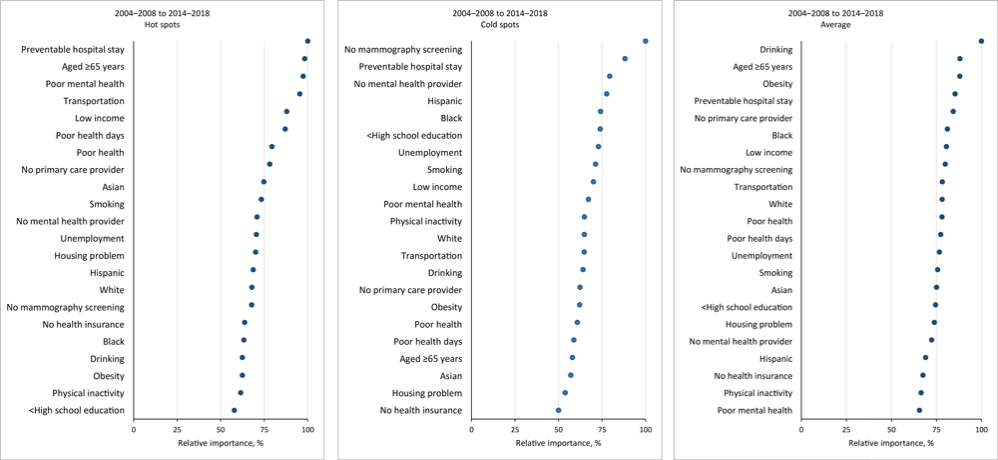
Relative importance of predictors of changes in total cancer mortality rates in hot spots and cold spots from geospatial analysis of US counties, 2004–2008 to 2014–2018.

The random forest analysis included 765 hot spot counties and 619 cold spot counties. Less than a high school education, preventable hospital stay, Asian race, and low income were the top 4 risk factors for change in total cancer mortality from 2004 through 2018 (Figure 2). For hot spots, the top 5 risk factors for changes in cancer mortality were preventable hospital stays, being aged 65 years or older, poor mental health, transportation issues, and low income (Figure 3). For cold spots, the top 5 risk factors were no mammography screening, preventable hospital stays, no mental health provider, Hispanic ethnicity, and Black race. For average regions, the top 5 risk factors were drinking, being aged 65 years or older, obesity, preventable hospital stays, and no primary care provider.

The patterns of risk factor importance in hot spots and cold spots (Figure 3) were similar to the patterns for all US counties. However, areas with the greatest increase in cancer death rates were associated with an older population and higher levels of alcohol consumption, and the greatest decreases in cancer death rates were associated with higher rates of mammography screening.

## Discussion

Although overall cancer death rates in the US are decreasing, disparities exist in how quickly they are decreasing across time and geographic areas. Our findings highlight similarities and differences in the hierarchy of risk factors for cancer death rates according to geographic hot spots and cold spots. Preventable hospital stays and mental health–related factors were top risks for cancer mortality risks in both hot spots and cold spots. These findings align with prior research showing that limited access to health care worsens outcomes, including preventable hospital stays and higher cancer death rates (23). Our study used a novel approach — machine learning (random forest analysis) — to analyze geospatial and temporal patterns in cancer death rates.

### Cancer mortality hot spots and cold spots

Using geospatial hot spot analysis, we found significant geospatial disparities in changes in total US cancer death rates between 2004–2008 and 2014–2018. The hot spots identified in our study have higher persistent cancer death rates compared with the national trend (rates decreased less than expected), while cold spots reflect areas with alleviated cancer death rates (rates decreased more than expected). Hot spots were predominantly concentrated in the Midwest, while cold spots were prevalent in the Southeast, Northeast, and 2 Mountain West states.

### Risk factors for cancer mortality hot spots and cold spots

We found various associations between negative socioeconomic position, access to health care services, and health behaviors. SDOH can positively or negatively influence health. We focused on negative SDOH and their influence on cancer mortality and how top risk factors of cancer mortality disparities differed in hot spots and cold spots. Some risk factors identified in our study were related to lower socioeconomic position, which can substantially affect access to cancer care. In general, people with low socioeconomic position have a heightened risk of various adverse health conditions, including cancer, due to factors such as unemployment, lower education levels, and poverty (23).

### Risk factors in hot spots

Preventable hospital stays are admissions that could have been avoided with adequate ambulatory care or health care coordination. Often caused by delayed or inadequate access, they can worsen health outcomes and increase mortality rates (24). Preventable hospitalization is common among cancer patients (25). Preventable hospitalizations are more prevalent in advanced-stage cancer than earlier-stage cancer, highlighting how inadequate health care access can worsen overall health outcomes, including death rates (26).

Our finding that being aged 65 years or older is a risk factor for cancer aligns with prior research linking age to preventable chronic conditions and cancer risk factors (27). The third top risk factor, poor mental health, mirrored research showing that mental health investment improves health outcomes, including lowering cancer death rates (28). In contrast, patients who develop mood, anxiety, or substance use disorders for the first time after a cancer diagnosis may be at an increased risk of cancer-related death (29).

Transportation availability, the fourth top risk factor in hot spots, is another factor that influences access to health care. Transportation problems can hinder health care in the US, especially for cancer patients, who have frequent health care visits, long treatment periods, and financial obligations (30). Transportation barriers can cause delays in follow-up care after abnormal screening test results and limit access to specialized oncology care (31). Overcoming transportation barriers is crucial for improving cancer care access and outcomes, particularly in areas with persistently high cancer death rates.

### Risk factors in cold spots

Preventable hospital stays and having no mental health provider were among the top 5 risk factors for changes in cancer mortality in cold spots. Having no mammography screening, being unemployed, and having less than a high school education were also in the top 5 risk factors. Our findings are consistent with a study across 79 countries that linked unemployment to higher mortality rates for cancers with available screening tests, suggesting the effect of economic instability on cancer outcomes (32).

Two other top risk factors for changes in cancer mortality in cold spots were Hispanic ethnicity and Black race. This finding demonstrates the effect of racial and ethnic health disparities on cancer mortality rates. Disparities in 5-year cancer survival persist between Black patients (67%) and White patients (72%), even among patients with similar income (27). Moreover, a study found that later-stage lung cancer was diagnosed more often in Black patients than in White patients even though Black patients had higher socioeconomic position (27). Lower overall cancer death rates among the Hispanic population might explain why Hispanic populations align with cold spots, but not hot spots. However, in a study that used data from 1950–2014, stomach and liver cancer death rates were higher in the Hispanic population than in the US general population; additionally, after adjustment for deprivation and other covariates, cancer death rates were significantly higher in the Hispanic and Black populations than in the non-Hispanic White population (33).

### Risk factors in average regions

Hot spots and cold spots depict extreme cancer disparities, while average regions reflect typical mortality trends. The study of average regions offers valuable insight into cancer disparities. Two of the top 3 risk factors associated with average regions were related to health behaviors (alcohol consumption and obesity), and the third was being aged 65 years or older, also a top risk factor in hot spots. Alcohol consumption, the top risk factor in average regions, increases cancer mortality risk in a dose-dependent manner (34). Older adults who are heavy drinkers have higher cancer death and incidence rates than nonheavy drinkers (35). Poor diet and low levels of physical activity are often associated with obesity. Excessive weight is linked to increased risk of various cancers (36). While the exact mechanisms for this connection are not known, it highlights the importance of healthy lifestyle habits in cancer prevention. Our findings underscore the need for interventions that promote healthy behaviors, particularly among older adults, to make strides in reducing cancer mortality (37).

The availability of cancer health care resources, which we did not examine due to data limitations, can also play a role in reducing cancer disparities. Cancer death rates in early Medicaid expansion states significantly decreased between 2007–2009 and 2012–2016 (25), highlighting the effect of policy on health care access. However, simply increasing access to health care is insufficient to eliminate disparities. Despite advancements in cancer treatment, cancer control at the population level requires resources to address SDOH and other risk factors. Our study used a machine learning algorithm, random forest analysis, to identify a set of SDOH and other factors that reflect the intricate interplay of individual, community, and societal influences on cancer disparities (7) and underscores the need for multilevel interventions, including comprehensive health policies, to address disparities effectively.

### Limitations and strengths

Our study has several limitations. First, our analysis focused on changes in total cancer death rates, which may not account for the various contributions of cancer types, such as lung cancer, a more prevalent cancer type with a strong modifiable risk factor of smoking. Second, our hot spot analysis identified areas with relatively high or low values, but it may not have fully considered the complex contextual factors influencing these patterns. Third, the missing county-level cancer death data and imputation to account for missing values may have affected the accuracy of our spatial analysis and are subject to interpretation. Fourth, aggregated cancer registry data protect patient privacy and can suppress differences and details in analysis, which may have affected our results. Nevertheless, hot spot analysis on imputed data provides a nuanced view of spatial patterns by incorporating estimated values for missing data. This approach can reveal potential clusters of extreme mortality rates that might be hidden by relying solely on cut points. Fifth, the study period predates the COVID-19 pandemic. Although future studies will include pandemic-era data, this study provides a valid framework for the utility of geospatial methods to study changes in cancer death rates. Lastly, while valuable, the application of random forest modeling is subject to its inherent limitations in providing insights into causality or the precise magnitude of variable effects on disparities in changes in total cancer death rates.

Despite these limitations, our study has several strengths. First, our geospatial analysis of total cancer death rates revealed areas with substantial disparities, highlighting the utility of geospatial methods in studying changes in cancer death rates. Geospatial analysis enabled a localized understanding of cancer mortality trends by visualizing and analyzing data across geographic space, revealing patterns and disparities that may not be evident from traditional statistical methods. Second, we used a machine learning algorithm to examine associated risk factors. This method excels in handling high-dimensional datasets and allowed us to capture intricate interactions among variables and mitigate overfitting, thus enhancing the robustness and generalization of our analysis.

### Conclusions

This study identified cancer mortality hot spots and cold spots and associated risk factors of cancer mortality between 2004–2008 and 2014–2018 at the US county level. Our findings emphasize the critical role of access to care, socioeconomic position, and health behaviors in reducing disparities in cancer death rates. Acknowledging these complexities and the various negative SDOH and demographic risk factors of cancer mortality by region, a comprehensive but localized approach that addresses both access to health care and the underlying SDOH is essential for achieving meaningful reductions in cancer disparities. This evidence informs public health practitioners and policymakers as they develop targeted interventions and policies. By understanding geospatial disparities in cancer and their underlying risk factors, public health can focus much-needed cancer treatment and prevention on the counties and populations most vulnerable to cancer-related death.
